# Association of the triglyceride-glucose index with immunotherapy efficacy and long-term prognosis in advanced Non-small cell lung cancer

**DOI:** 10.3389/fonc.2025.1616302

**Published:** 2025-09-26

**Authors:** Lijie Ma, Wanling Zhu, Yulang Wang, Li Zhao, Xiangju Wei, Xianmin Xiong, Guijuan Ji

**Affiliations:** ^1^ Xuzhou Medical University, Xuzhou, China; ^2^ Fourth Affiliated Hospital, School of Medicine, Zhejiang University, Yiwu, China; ^3^ The Affiliated Hospital of Xuzhou Medical University, Xuzhou, China

**Keywords:** non-small cell lung cancer, TyG index, pembrolizumab monoclonal antibody, efficacy, safety

## Abstract

**Background:**

Non-small cell lung cancer (NSCLC), the most common subtype of primary lung cancer, represents a major component of the global cancer burden. Despite the availability of several immunotherapeutic and immune-combination treatment modalities, its prognosis remains poor. Therefore, it is particularly important to predict the prognosis of these patients.

**Aim:**

To investigate whether a marker of insulin resistance [the triglyceride-glucose (TyG) index] could predict clinical outcomes in patients with advanced non-small cell lung cancer receiving pembrolizumab and chemotherapy.

**Methods:**

We retrospectively analyzed data of 243 patients with advanced non-small cell lung cancer treated with pembrolizumab and chemotherapy, dividing them into high and low TyG index groups based on an optimal cut-off value of 1.61, determined using the X-tile software. Cox proportional hazards regression analysis identified independent prognostic factors for overall survival (OS), and a prediction nomogram model was developed based on them.

**Results:**

The study cohort included 132 patients with a high TyG index and 111 with a low TyG index. The median progression-free survival and OS in the low TyG index group were significantly longer than in the high TyG index group. The objective response rate was 45.05% in the low TyG index group and 25.76% in the high TyG index group, while the respective disease control rates were 85.59% and 53.03%. Multifactorial regression analysis identified pre-treatment maximum tumor diameter, PD-L1 TPS (Tumor Proportion Score), BMI(Body Mass Index), and TyG index as independent prognostic predictors of OS. The nomogram model emphasized the importance of the TYG index as a major prognostic predictor, followed by the PD-L1 TPS score, pre-treatment tumor diameter, and BMI.

**Conclusion:**

The TyG index is a long-term predictor of the efficacy of combined immunotherapy and chemotherapy in patients with advanced NSCLC. Patients with a low TyG index have a better prognosis.

## Introduction

Primary lung cancer is the most common cancer and the leading cause of cancer-related death worldwide (1). Non-small cell lung cancer (NSCLC) is the predominant form of lung cancer. Despite advances in NSCLC treatment, patients are often diagnosed at an advanced stage, missing the optimal time for surgery (2). Therefore, non-surgical treatment options such as immunotherapy are crucial when treating NSCLC (3). It has been shown that the binding of programmed death ligand 1 (PD-L1) to its receptor (PD-1) participates in the immune escape of tumor cells as it inhibits the proliferation and cytokine production of activated T lymphocytes (4).

Pembrolizumab, an immune checkpoint inhibitor, blocks PD-1/PD-L1/PD-L2 interactions by immune suppression and T cell activation to enhance antitumor responses (5, 6). Several studies have shown that programmed cell death ligand inhibitors, in combination with chemotherapeutic agents, significantly improve the outcomes of patients with unresectable NSCLC (7–9). Combination therapy for advanced NSCLC that uses immunosuppressants and chemotherapeutic agents can inhibit tumor growth and enhance the body’s immune function through a dual mechanism, improving survival rates and the quality of life (10). Therefore, the combination of PD-1/PD-L1 inhibitors and chemotherapy has been approved as a preferred treatment modality for patients with advanced NSCLC.

The triglyceride-glucose (TyG) index assesses the metabolic status. It is the product of serum triglyceride and glucose levels. The index is considered a reliable surrogate biomarker of insulin resistance (11). Studies have found that the TyG index is a potential marker for type 2 diabetes, colorectal cancer, breast cancer, and liver cancer (12, 13). It was also shown that insulin resistance promotes tumor growth and metastasis by altering inflammation levels and the immune response and impairing effector immune cell functions, including cytotoxic T lymphocytes and natural killer cells, further affecting the efficacy of tumor immunotherapy (14). A study on prostate cancer noted that the higher survival rate in patients with a low TyG index might be associated with downregulation of specific oncogenes and/or upregulation of PD-1 expression (15). However, few studies have assessed the TyG index as a prognostic predictor in patients with advanced NSCLC treated with immunosuppressive agents in combination with chemotherapy. This knowledge gap is critical in NSCLC, as insulin resistance might synergize with PD-1/PD-L1 blockade as a resistance mechanism (16). Therefore, we investigated the prognostic value of the TyG index in patients with advanced NSCLC receiving chemoimmunotherapy.

## Materials and methods

### Patient characteristics

The clinical data of patients with NSCLC admitted to the Affiliated Hospital of Xuzhou Medical University between December 2022 and October 2024 were retrospectively analyzed. Patients who met the following criteria were included in the study: (1) diagnosed with advanced lung cancer by pathology and imaging; (2) received pembrolizumab in combination with chemotherapy and have data on pre-treatment biochemical and hematological indices; (3) received at least three treatment cycles; (4) had at least one measurable tumor; (5) aged 18 to 70 years when treated; (6) had an Eastern Oncology Cooperative Oncology Group (ECOG) score ≤1; (7) had complete clinical follow-up data.

**Figure 1:**
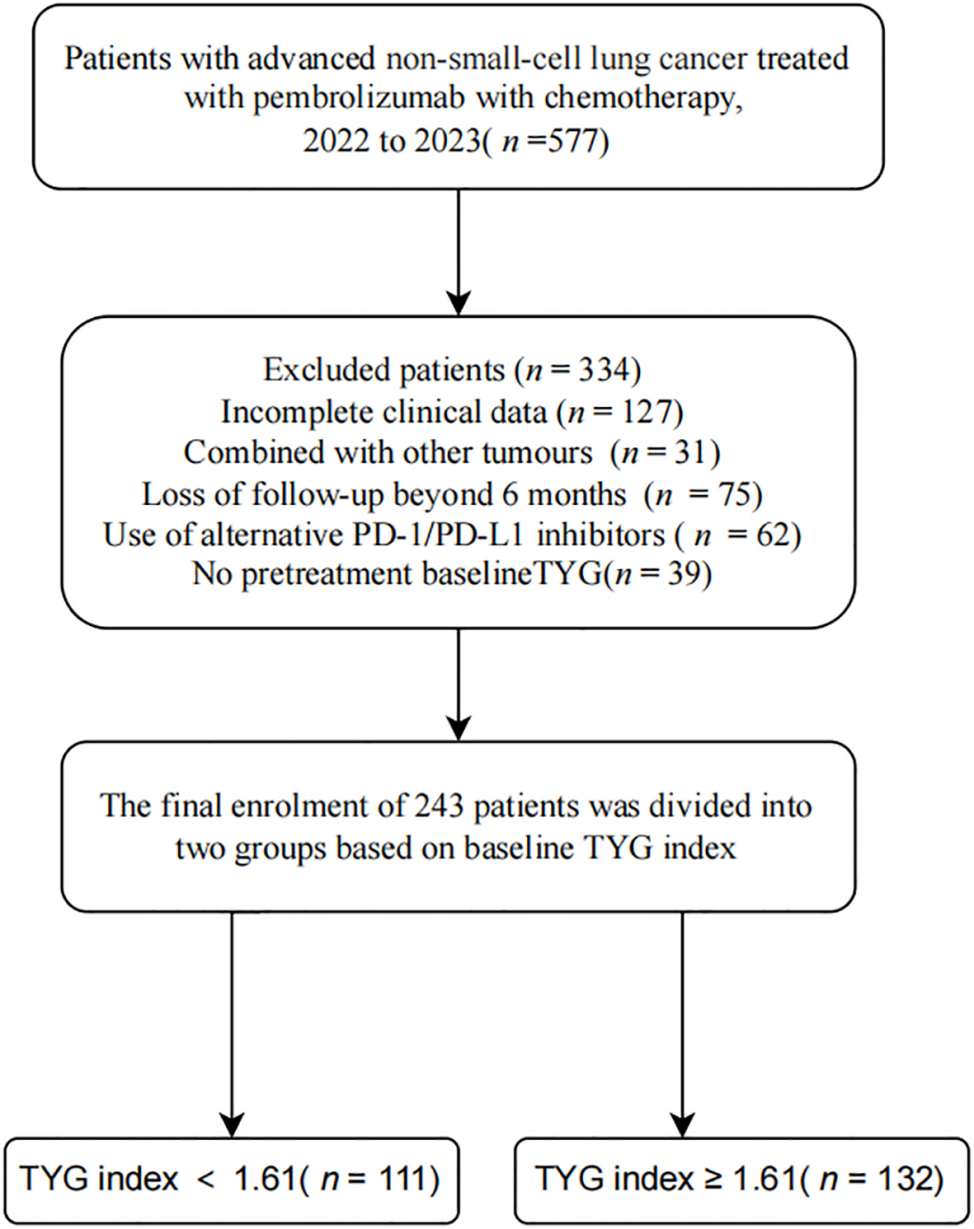
Flow chart depicting the screening of NSCLC patients who received pembrolizumab combined with chemotherapy. PD-1, Programmed cell death protein 1; PD-L1, Programmed cell death ligand 1; TyG, Triglyceride-glucose.

Exclusion criteria: (1) received previous treatment with PD-L1 inhibitors or targeted drugs; (2) had severe infections or systemic inflammation; (3) had also hematological or immune system disorders, hepatic insufficiency, or hepatitis; (4) had a history of long-term steroids or immunosuppressant use; (5) had severe cardiac, hepatic, renal, or other organic pathologies; (6) had also other malignant tumors or non-primary NSCLC; (7) inaccurate or incomplete clinical data. Patients who received lipid-modifying drugs (e.g., statins and betas) during the study period were excluded from the analyses to reduce potential confounding effects on the TyG index. Based on the inclusion and exclusion criteria, the study included 243 patients (**Figure 1**). The Ethics Review Committee of the Affiliated Hospital of Xuzhou Medical University approved the study (XYFY-), which adhered to the principles outlined in the Declaration of Helsinki. The requirement for written informed consent was waived due to the retrospective nature of the study.

### Definition of the TYG index

The TyG index was calculated as follows: TyG index (mmol/L) = ln [fasting triglycerides (mmol/L) × fasting glucose (mmol/L)/2]. Fasting triglyceride and glucose levels were determined by an enzymatic method using an automated biochemical analyzer (AU2700, Olympus). The optimal TyG index cut-off value was calculated by the X-tile software using the survival time after the combined immunochemotherapy.

### Grouping and treatment programs

The patients were divided into high (≥1.61) and low (<1.61) TyG index groups based on the calculated cut-off value. Pembrolizumab (200 mg) was administered intravenously once every three weeks. The chemotherapy regimen comprised 260 mg/m^2^ intravenous paclitaxel (albumin-based) once every three weeks and 75 mg/m² cisplatin. Patients with non-squamous histology received 500 mg/m^2^ pemetrexed once every three weeks and 75 mg/m² cisplatin.

### Assessment of efficacy

Patients underwent chest CT scanning every cycle and an enhanced CT or MRI examination every two cycles. The efficacy of treatment was assessed by applying the solid tumor efficacy evaluation criteria (RECIST version 1.1) and was classified into complete remission (CR), partial remission (PR), stable disease (SD), and disease progression (PD). Objective response rate (ORR) = (CR+PR)/total number of cases × 100%; Disease control rate (DCR) = (CR+PR+SD)/total number of cases × 100%. Progression-free survival (PFS) was defined as the time from the start of the 1st immunotherapy to tumor progression or death. Overall survival (OS) was defined as the time from the start of treatment to death from any cause (event). Survival time was calculated in months. Long-term treatment effects were assessed using OS and PFS. To ensure accuracy, all imaging data were evaluated by two independent radiologists with more than 10 years of experience. In cases of disagreement, a third radiologist with more than 10 years of experience assessed the information to help reach consensus and minimize inter-observer error. The final follow-up visit was in October 2024.

### Variables included in the study and follow-up

TYG index and tumor response were assessed by blinded independent reviewers at baseline and after three cycles of pembrolizumab plus platinum-based chemotherapy. PFS and OS were defined as primary endpoints. Follow-up was conducted through scheduled hospital visits or telephone interviews every 3 weeks, with a final data collection cutoff date of October 2024.

### Data analysis

Data were analyzed using R software (version 4.4.1). The Kolmogorov-Smirnov test was used to assess normality prior to hypothesis testing for continuous variables. Continuous variables are presented as the mean ± SD, and categorical variables are described using frequencies (percentages). Normally distributed continuous data are expressed as means ± standard deviations (SD) (with Welch’s correction if variances were unequal based on Levene’s test), while non-normally distributed continuous data are expressed as medians with interquartile ranges (IQR). The baseline characteristics between groups were compared using the Fisher’s exact test, *χ* ^2^ test, and *t*-test. The Kaplan-Meier method was used to calculate the median OS and PFS, which were used in the X-tile procedure to determine the optimal survival-related cut-off value for TyG. Cox proportional hazards regression was used for univariate and multivariate analyses. Multivariate analysis used stepwise backward selection based on the Akaike Information Criterion (AIC), with entry and removal thresholds of *P*<0.05 and *P* > 0.10, respectively. A nomogram was developed to predict the survival at 12, 24, and 36 months. The variables were selected according to their statistical significance and clinical relevance and weighted according to their hazard ratio (HR) derived from multivariate analysis. Internal validation of the model and the accuracy of the predictions were assessed by the bootstrap method with 1,000 resamples. Predictions generated by the model were compared with actual results to assess their calibration and reduce overfitting. All statistical tests were two-sided, and *P*<0.05 was considered statistically significant.

## Results

### Patient characteristics

This retrospective study analyzed the data of 243 patients with advanced NSCLC who received pembrolizumab in combination with platinum-based chemotherapy between December 2022 and October 2024 at Xuzhou Medical University Hospital (**Figure 1**). Among them, 111 were classified into the low TyG (<1.61) index group and 132 into the high TyG (≥1.61) index group. We excluded 334 patients because of incomplete documentation of clinical data (*n*=127), comorbid with other malignancies or non-primary NSCLC (*n*=31), loss to follow-up for more than six months (*n*=75), used alternative PD-1/PD-L1 inhibitors or targeted medications (*n*=62), and missing baseline TyG index data (*n*=39). **Table 1** provides a comprehensive overview of patient characteristics, including clinicopathological features such as age, sex, BMI, smoking and drinking status, ECOG(Eastern Cooperative Oncology Group) score, history of COPD(Chronic Obstructive Pulmonary Disease), maximum pre-treatment tumor diameter, extrapulmonary invasion, and various laboratory parameters such as fasting blood glucose, fasting triglycerides, and albumin levels. The two groups differed significantly in BMI, TYG, PD-L1 expression level, and tumor diameter. In this study, 243 NSCLC patients were stratified by histological subtype. The majority had adenocarcinoma (n = 116, 47.7%) or squamous cell carcinoma (n = 107, 44.0%). Less common subtypes included large cell carcinoma (n = 9, 3.7%), adenosquamous carcinoma (n = 6, 2.5%), and sarcomatoid carcinoma (n = 5, 2.1%). This distribution aligns with established epidemiological patterns of NSCLC.

**Table 1:** Baseline patient characteristics, comparing the low (<1.61) and high (≥1.61) triglyceride-glucose index groups.

Variable	Overall (*n*=243)	Low TyG index (*n*=111)	High TyG index (*n*=132)	*P*-value
BMI, kg/m^2^, mean ± SD	22.78 ± 2.93	22.12 ± 2.91	23.42 ± 2.76	<0.001
Fasting triglycerides, mmol/L, mean ± SD	1.39 ± 0.66	1.19 ± 0.57	1.56 ± 0.69	<0.001
Fasting glucose, mmol/L, mean ± SD	5.75 ± 1.68	5.48 ± 1.24	5.97 ± 1.95	0.018
TyG index, mean ± SD	1.78 ± 0.51	1.41 ± 0.44	2.09 ± 0.32	<0.001
Age, years	64.18 ± 8.74	63.06 ± 8.52	65.11 ± 8.84	0.068
Sex				0.470
Female	54 (22.22)	27 (24.32)	27 (20.45)	
Male	189 (77.78)	84 (75.68)	105 (79.55)	
Drinking history				0.862
No	165 (67.90)	76 (68.47)	89 (67.42)	
Yes	78 (32.10)	35 (31.53)	43 (32.58)	
Smoking history				0.787
No	105 (43.21)	49 (44.14)	56 (42.42)	
Yes	138 (56.79)	62 (55.86)	76 (57.58)	
ECOG				0.336
0	143 (58.85)	69 (62.16)	74 (56.06)	
1	100 (41.15)	42 (37.84)	58 (43.94)	
COPD				0.480
No	170 (70.2)	75 (67.4)	94 (71.4)	
Yes	72 (29.8)	36 (32.6)	38 (28.6)	
Histological type				0.604
Adenocarcinoma	116 (47.74)	55 (49.55)	61 (46.21)	
Other	127 (52.26)	56 (50.45)	71 (53.79)	
CA-125, U/mL				0.427
<35	103 (42.39)	44 (39.64)	59 (44.70)	
≥35	140 (57.61)	67 (60.36)	73 (55.30)	
CEA, ng/mL				0.891
<3	128 (52.67)	59 (53.15)	69 (52.27)	
≥3	115 (47.33)	52 (46.85)	63 (47.73)	
PD-L1 expression				0.046
TPS < 50%	112 (46.09)	44 (39.64)	68 (51.52)	
TPS ≥ 50%	131 (53.91)	67 (60.36)	64 (48.48)	
Tumor diameter, mm				0.025
<50	141 (58.02)	73 (65.77)	68 (51.52)	
≥50	102 (41.98)	38 (34.23)	64 (48.48)	
Extrapulmonary metastasis				0.111
No	131 (53.91)	66 (59.46)	65 (49.24)	
Yes	112 (46.09)	45 (40.54)	67 (50.76)	

### Tumor response

**Table 2** describes the efficacy of the response to the oncological treatment. No patient achieved CR, 84 achieved PR, 81 achieved SD, and 78 showed PD. The ORR was 45.05% and 25.76% in the low and high TyG index groups, respectively (*P*=0.002), and the DCR was 85.59% and 53.03%, respectively (*P*<0.001).

**Table 2:** Tumor responses in the low (<1.61) and high (≥1.61) triglyceride-glucose index groups, *n* (%).

Variable	Low TyG index (*n*=111)	High TyG index (*n*=132)	*χ* ^2^	*P*-value
PD	50 (45.05)	34 (25.76)		
SD	45 (40.54)	36 (27.27)		
PR	16 (14.41)	62 (46.97)		
ORR			9.92	0.002
Yes	61 (54.95)	98 (74.24)		
No	50 (45.05)	34 (25.76)		
DCR			29.32	<0.001
Yes	16 (14.41)	62 (46.97)		
No	95 (85.59)	70 (53.03)		

### PFS and OS

**Figure 2:**
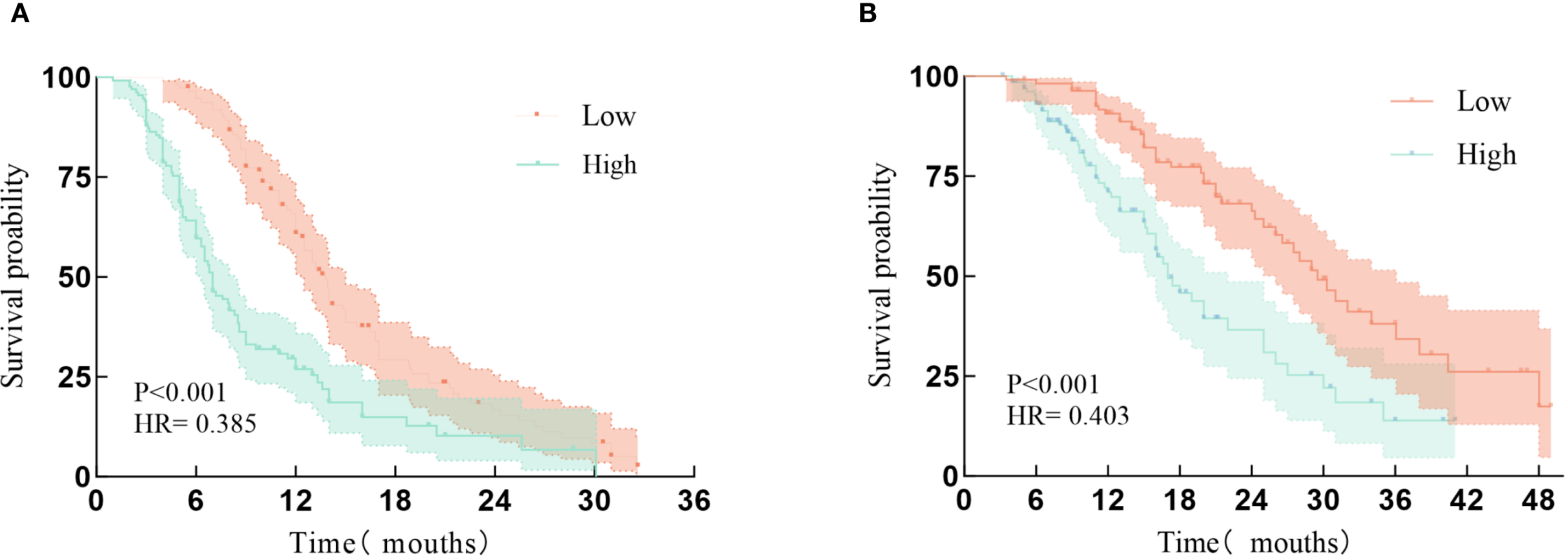
The association of low and high triglyceride-glucose indexes and the long-term prognosis of patients with advanced NSCLC. **(A)** Kaplan-Meier plot of progression-free survival in the triglyceride-glucose (TyG) < 1.61 and ≥ 1.61 index groups; **(B)** Kaplan-Meier plot of overall survival in the TyG < 1.61 and ≥ 1.61 index groups. HR, Hazard ratio.

The median PFS in the low TyG index group was significantly longer than in the high TyG index group [13.8 (9.2–14.4) vs. 8.2 (7.3–8.7) months; *P*<0.001; HR, 0.385; 95% CI, 0.278–0.534; **Figure 2A**]. The median OS in the low TyG index group was significantly longer than in the high TyG group [26.1 (23.4–29.8) vs. 17.0 (13.9–18.3) months; *P*<0.001; HR, 0.403; 95% CI, 0.268–0.608; **Figure 2B**].

### Univariate and multifactorial analyses of PFS and OS

**Figure 3:**
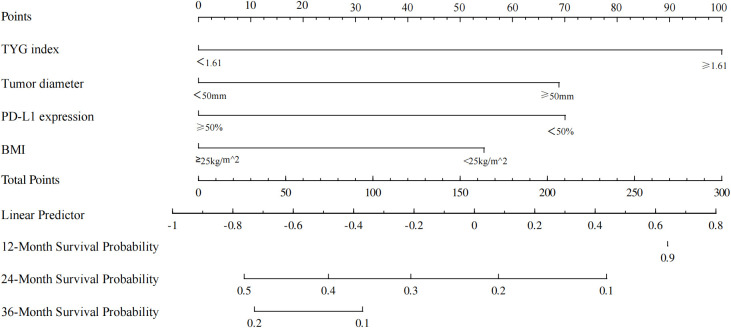
Graph depicting the nomogram model for predicting 12-, 24-, and 36-month overall survival. BMI, Body mass index; TyG, Triglyceride-glucose; PD-L1, Programmed cell death ligand 1; OS, Overall survival.

**Table 3** presents the results of univariate and multivariate Cox proportional hazards regression analyses for PFS. Multivariate regression analysis revealed several independent prognostic predictors for PFS in patients with NSCLC. Patients with a higher ECOG score had a shorter PFS (HR, 1.24; 95% CI, 1.12–1.55; *P*=0.021). **Table 4** presents items related to OS. Good metabolic function was strongly correlated with long OS, with a lower TyG index indicating better metabolic function, specifically in the benign prognostic factors of PFS (HR, 4.05; 95% CI, 2.84–5.31; *P*<0.001) and OS (HR, 2.87; 95% CI, 1.43–4.52; *P*=0.003), suggesting that patients with better metabolic function responded more favorably to the treatment. Higher PD-L1 expression in tumor cells (TPS ≥ 50%) predicted longer PFS (HR, 0.51; 95% CI, 0.34–0.78; *P*=0.004) and OS (HR, 0.66; 95% CI, 0.44–0.89; *P*=0.011). Although BMI was an independent predictor for OS (HR, 0.78; 95% CI, 0.29–0.86, *P*=0.029), it did not predict PFS in the multivariate regression analysis (*P*=0.142), implying that higher BMI was associated with improved long-term survival but not with short-term treatment tolerance and PFS. Elevated CEA levels (≥3 ng/mL) predicted shorter PFS (HR, 1.21, 95% CI 0.94–1.81, *P*=0.039). Possibly due to overlapping effects with more influential factors such as ECOG score and PD-L1 expression, age, sex, extrapulmonary metastases, and pathological staging were insignificant predictors in the multivariate regression analysis. The nomogram model developed for 12, 24, and 36 months based on the independent prognostic predictors obtained from the multivariate regression analysis showed a consistency index of 0.772 for the training set and 0.786 for the validation set (**Figure 3**), considered to have good predictive properties.

**Table 3:** Univariate and multivariate regression analyses of prognostic predictors for progression-free survival.

Variable	Univariate		Multivariate	
	HR (95% CI)	*P*	HR (95% CI)	*P*
Age (≥ 60 vs < 60 years)	0.85 (0.59–1.26)	0.410		
TyG (high vs low)	4.81 (3.32–6.98)	<0.001	4.05 (2.84–5.31)	<0.001
Smoking history (yes vs no)	1.08 (0.78–1.50)	0.658		
Drinking history (yes vs no)	1.20 (0.86–1.69)	0.284		
Sex (male vs female)	1.31 (0.88–1.96)	0.186		
Tumor diameter (≥ 50 vs < 50mm)	1.26 (0.91–1.75)	0.170		
CEA (< 3 vs ≥ 3 ng/mL)	1.38 (1.00–1.91)	0.052	1.21 (0.94–1.81)	0.039
BMI (< 25 vs ≥ 25 kg/m^2^)	0.71 (0.51–0.98)	0.039	0.79 (0.59–1.03)	0.142
PD-L1 expression (TPS < 50% vs TPS ≥ 50%)	0.45 (0.31–0.64)	<0.001	0.51 (0.34–0.78)	0.004
ECOG score (0 vs 1)	1.39 (1.00–1.93)	0.017	1.24 (1.12–1.55)	0.021
COPD (yes vs no)	1.15 (0.69–1.93)	0.593		
Histological type (others vs adenocarcinoma)	1.38 (1.00–1.92)	0.052	1.16 (0.97–1.73)	0.113
Extrapulmonary metastasis (yes vs no)	1.29 (0.93–1.78)	0.125		
Fasting triglycerides	1.20 (0.94–1.53)	0.139		
Fasting glucose	0.99 (0.90–1.10)	0.916		
CA-125 (< 35 vs ≥ 35 U/mL)	0.95 (0.68–1.32)	0.794		

**Table 4:** Univariate and multivariate analyses of prognostic predictors for overall survival.

Variable	Univariate		Multivariate	
	HR (95% CI)	*P*	HR (95% CI)	*P*
Age (≥ 60 vs < 60 years)	1.00 (0.98 ~ 1.03)	0.770		
TyG (high vs low)	3.19 (1.88 ~ 5.41)	<.001	2.87 (1.19 ~ 4.89)	0.003
Smoking history (yes vs no)	1.61 (0.97 ~ 2.68)	0.067	1.75(1.02–2.73)	0.123
Drinking history (yes vs no)	0.66 (0.37 ~ 1.17)	0.157		
Sex (male vs female)	1.05 (0.59 ~ 1.85)	0.871		
Tumor diameter (≥ 50 vs < 50mm)	2.24 (1.36 ~ 3.68)	0.001	1.51 (1.08–2.15)	0.015
CEA (< 3 vs ≥ 3 ng/mL)	1.09 (0.66 ~ 1.78)	0.740		
BMI (< 25 vs ≥ 25 kg/m^2^)	0.52 (0.31 ~ 0.86)	0.010	0.78 (0.29–0.86)	0.029
PD-L1 expression (TPS < 50% vs TPS ≥ 50%)	0.55 (0.34 ~ 0.71)	0.019	0.66 (0.44–0.89)	0.011
ECOG score (0 vs 1)	1.32 (0.80 ~ 2.18)	0.283		
COPD (yes vs no)	0.83(0.487~1.403)	0.480		
Histological type (others vs adenocarcinoma)	1.31 (0.81 ~ 2.14)	0.271		
Extrapulmonary metastasis	1.16 (0.71 ~ 1.89)	0.552		
Fasting triglycerides	1.20 (0.84 ~ 1.73)	0.316		
Fasting glucose	1.10 (0.98 ~ 1.24)	0.112		
CA-125 (< 35 vs ≥ 35 U/mL)	1.00 (1.00 ~ 1.00)	0.851		

### Validation of the prognostic model

**Figure 4:**
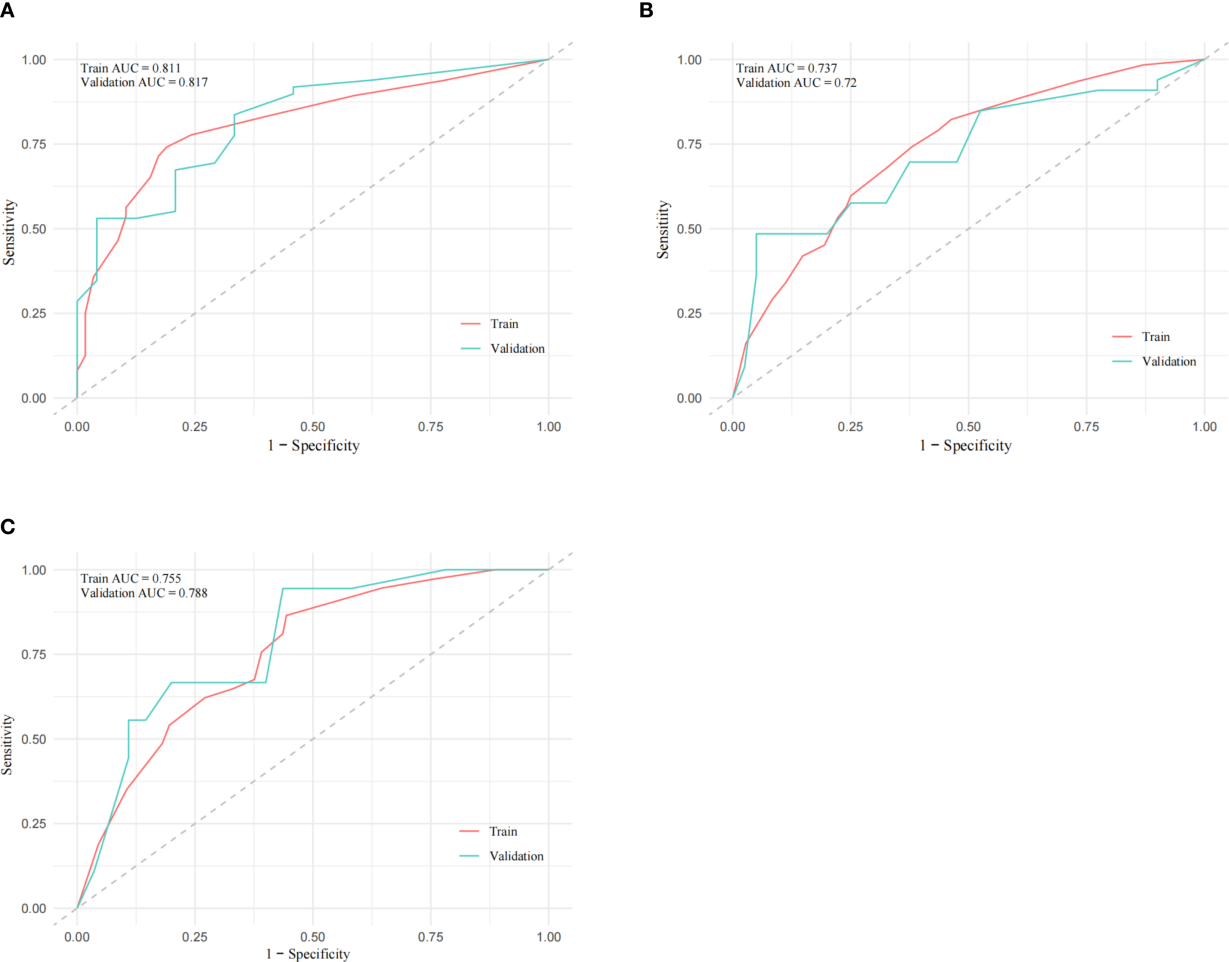
Receiver operating characteristic evaluation plots for the prognostic model. A: Receiver operating characteristic evaluation plots for the training and internal validation sets using the prognostic prediction model to predict the 12-month **(A)**, 24-month **(B)**, and 36-month **(C)** outcomes. AUC, Area under the curve.

**Figure 5:**
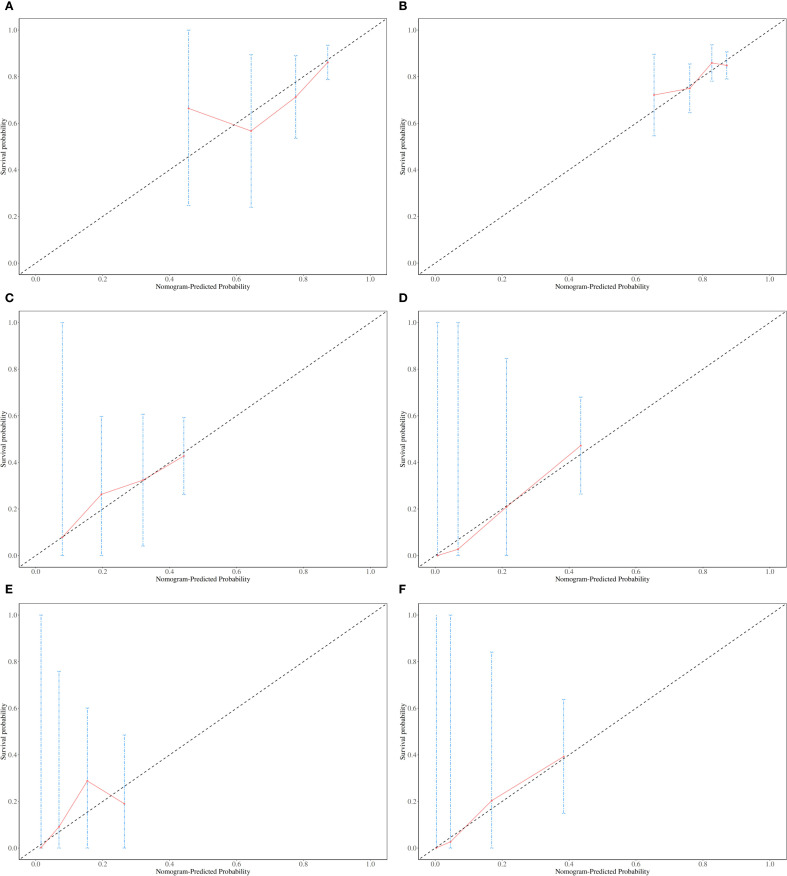
Calibration plots for the nomogram model. **(A)** A calibration plot for overall survival (OS) in the training set at 12 months; **(B)** A calibration plot for OS in the internal validation set at 12 months; **(C)** A calibration plot for OS in the training set at 24 months; **(D)** A calibration plot for OS in the internal validation set at 24 months; **(E)** A calibration plot for OS in the training set at 36 months; **(F)** A calibration plot for OS in the internal validation set at 36 months.

The patients were randomly assigned to two groups at a ratio of 7:3. We assigned 170 cases to the training set and 70 to the internal validation set. **Table 5** lists the baseline characteristics of these two sets. The respective OS AUCs(Area Under Curve) for the training and internal validation sets were 0.811 and 0.817 for 12 months (**Figure 4A**), 0.737 and 0.720 for 24 months (**Figure 4B**), and 0.755 and 0.788 for 36 months (**Figure 4C**). The OS calibration curves for the training and internal validation sets at 12, 24, and 36 months are shown in **Figure 5**. These curves indicate that the risk predicted by the model is in close agreement with the actual observed risk.

**Table 5:** Comparison of features between the training and internal validation sets, *n* (%).

Variable	Test (*n*=73)	Training (*n*=170)	χ²	*P*-value
Age, years			1.94	0.164
< 60	37 (51.11)	74 (42.43)		
≥ 60	36 (48.89)	96 (57.67)		
Sex			0.01	0.940
Female	16 (21.92)	38 (22.35)		
Male	57 (78.08)	132 (77.65)		
Smoking history			0.02	0.897
No	32 (43.84)	73 (42.94)		
Yes	41 (56.16)	97 (57.06)		
Drinking history			0.53	0.466
No	52 (71.23)	113 (66.47)		
Yes	21 (28.77)	57 (33.53)		
ECOG score			2.05	0.152
0	48 (65.75)	95 (55.88)		
1	25 (34.25)	75 (44.12)		
COPD			0.498	0.311
No	49 (67.4)	121 (71.4)		
Yes	24 (32.6)	49 (28.6)		
BMI, kg/m^2^			0.04	0.833
<25	44 (60.27)	100 (58.82)		
≥25	29 (39.73)	70 (41.18)		
Histological type			2.08	0.149
Adenocarcinoma	40 (54.79)	76 (44.71)		
Other	33 (45.21)	94 (55.29)		
CA-125, U/mL			5.06	0.403
<35	23 (31.51)	80 (47.06)		
≥35	50 (68.49)	90 (52.94)		
CEA, ng/mL			1.62	0.203
<3	43 (58.90)	85 (50.00)		
≥3	30 (41.10)	85 (50.00)		
PD-L1 expression			0.03	0.856
TPS<50%	33 (45.21)	79 (46.47)		
TPS≥50%	40 (54.79)	91 (53.53)		
Tumor diameter, mm			0.21	0.644
<50	41 (56.16)	90 (52.94)		
≥50	32 (43.84)	80 (47.06)		
Extrapulmonary metastasis			1.53	0.217
No	38 (52.05)	103 (60.59)		
Yes	35 (47.95)	67 (39.41)		

## Discussion

This study analyzed the association of the TyG index and the efficacy of immunotherapy combined with chemotherapy in patients with NSCLC, and a predictive model of long-term prognosis was constructed. The results showed that the low TyG index group had better median OS, PFS, ORR, and DCR than the high TyG group. The independent prognostic predictors for PFS were the ECOG score, pre-treatment tumor maximal diameter, and TyG index. The independent prognostic predictors for OS were PD-L1 expression, pre-treatment tumor maximal diameter, BMI, and TyG index.

NSCLC is the most common pathological subtype of lung cancer, accounting for 80–85% of all cases. Immune checkpoint inhibitors (ICIs) such as anti-PD-1 and PD-L1 are standard therapies for advanced NSCLC (17). Pembrolizumab, a PD-1 inhibitor, can relieve PD-1 pathway-mediated immunosuppression and activate T lymphocytes to exert anti-tumor effects (18). Immunotherapy or combination therapy for patients with NSCLC has been further optimized following the wide application of immune drugs; However, relevant studies predicting the long-term prognosis of these patients remain lacking (19). The TyG index has received increasing attention in recent years, but most related studies have focused on patients with cardiovascular disease and metabolic abnormalities (20, 21). This study introduced the TyG index into combined immunotherapy and chemotherapy for NSCLC. We found that a lower TyG index was associated with a better prognosis for patients with NSCLC receiving such a combination therapy. Previous prognostic studies found similar associations in patients treated for pancreatic cancer and renal cell carcinoma (22, 23). High TyG index (reflecting insulin resistance) is associated with cholesterol ester accumulation in CD8+ T cells through the upregulation of the sterol regulatory element-binding protein (SREBP) pathway, inducing endoplasmic reticulum stress and PD-1 overexpression. Concurrently, this metabolic dysregulation impairs natural killer cell cytotoxicity through mTOR-dependent metabolic exhaustion (24). Insulin resistance can disturb the balance of T cell subsets, which is marked by a decrease in CD4^+^ T cell counts and an increase in CD8^+^ T cells (25). This upsets the coordination of immune responses and weakens anti-tumor immune reactions. What’s more, insulin resistance lowers the expression of chemokine receptors on T cells. This affects how T cells move to tumor sites and their interactions with tumor cells and other immune cells, in turn restricting the effectiveness of immunotherapy (26). In other words, patients with low TyG index usually have low cholesterol levels, suppressing the expression of immunosuppressive factors in the tumor microenvironment. PD-L1 expression is an important predictor of ICI efficacy. A lower TyG index might be associated with improved metabolic and immune status of patients with PD-L1 positivity, enhancing their response to immunotherapy (27, 28). Notably, the synergistic effect of low TyG index and PD-L1 positivity could be mediated through metabolic reprogramming of tumor-associated macrophages (TAMs). Preclinical evidence indicates that insulin sensitivity reduces M2-polarized TAM infiltration, thereby enhancing PD-L1 inhibitor response. Chronic inflammation with a high TyG index should not be overlooked, as a high TyG index reflects insulin resistance, dyslipidemia, and impaired glucose metabolism, which are usually accompanied by elevated systemic inflammation levels (29). Previous studies have shown that the tumor state is associated with chronic inflammation and that increased levels of inflammation are often associated with a poorer prognosis. High levels of inflammation elevate the inflammatory factors (e.g., TNF-α IL-6, CRP, etc.), further limiting immune cell recovery and the effects of anti-angiogenic drugs (30, 31). Obesity tends to go hand in hand with high TyG indices, yet it was previously suggested in a retrospective study of patients with melanoma and NSCLC that patients with obesity have a better prognosis than those with a lower BMI when treated with ICIs (32, 33). This discrepancy might be due to differences in patient cohorts (e.g., inclusion of PD-L1-negative subgroups), chemotherapy regimens, study populations, and treatment modalities. Furthermore, BMI has some limitations, mainly in its inability to explain the interference of metabolic syndrome and chronic inflammation in the efficacy of immunotherapy (34). Therefore, the TyG index is an advantageous bio-predictor.

In this study, multivariate Cox proportional hazards regression analysis showed that BMI, pre-treatment tumor diameter, PD-L1 TPS score, and the TyG index were independent prognostic predictors for OS. Subsequently, we developed a column-line diagram model integrating these prognostic predictors. The results showed that the TyG index had the greatest explanatory power for OS, followed by the PD-L1 TPS score, pre-treatment tumor diameter, and BMI. The C-index was calculated, and calibration curves were generated to assess the validity of the nomogram prediction model. The model indicated a C-index of 0.776 for the internal validation set. The calibration curve showed that the model was in good agreement with the actual data, confirming its reliability and accuracy.

This study had several limitations. First, it was a single-center retrospective study. Our findings should be validated in a multicenter prospective study. The characteristics of a single-center retrospective study and the limited sample prevented us from performing external validation. Future studies need to be externally validated using data from different populations and centers to improve the generalizability and reliability of the model. Our findings should be interpreted considering the exclusion of 58% of screened patients, predominantly due to incomplete data. While this may affect generalizability, our sensitivity analyses support result robustness. Future multi-center studies should implement real-time data monitoring to minimize exclusions. Second, we chose a combination of pembrolizumab and chemotherapy, which reduced treatment bias and strengthened our grading; however, it means that our conclusions should be further validated for other ICIs. Third, patients receiving drugs affecting lipid metabolism and those with complex comorbidities were excluded from the study, limiting the applicability of our results to a wider range of patients with NSCLC. Fourth, this study used a statistical method to determine the optimal cut-off value for the TyG index; however, this criterion might be affected by factors such as sample size and the study population characteristics (e.g., race, age, and basal metabolic status), and might not be applicable to other populations or medical settings. Furthermore, the formulas used to calculate the TyG index and the cut-off value might differ across studies, further limiting the feasibility of cross-study comparisons and generalization of results. Dynamic monitoring of the TyG index throughout the treatment course is recommended. A persistently elevated or progressively rising TyG level may function as an early warning signal, prompting clinicians to appraise the efficacy of the current therapeutic regimen. This facilitates timely adjustments—such as refining chemotherapy combinations or integrating adjuvant therapies like anti-angiogenic agents—thereby enabling personalized treatment management. In the future, larger, multicenter studies are needed to validate the stability and generalizability of the cut-off value and explore the existence of stratified dynamic cut-off values to better guide clinical decision-making.

## Data Availability

The datasets presented in this article are not readily available because Data may involve patient privacy. Requests to access the datasets should be directed to malijie61@163.com.
